# Inhibition of the α-carbonic anhydrase from *Vibrio cholerae* with amides and sulfonamides incorporating imidazole moieties

**DOI:** 10.1080/14756366.2017.1327522

**Published:** 2017-06-01

**Authors:** Daniela De Vita, Andrea Angeli, Fabiana Pandolfi, Martina Bortolami, Roberta Costi, Roberto Di Santo, Elisabetta Suffredini, Mariangela Ceruso, Sonia Del Prete, Clemente Capasso, Luigi Scipione, Claudiu T. Supuran

**Affiliations:** a Dipartimento di Chimica e Tecnologie del Farmaco, Istituto Pasteur-Fondazione Cenci Bolognetti, Università di Roma La Sapienza, Roma, Italy;; b Dipartimento Neurofarba, Sezione di Scienze Farmaceutiche e Nutraceutiche, Università degli Studi di Firenze, Florence, Italy;; c Dipartimento di Sicurezza Alimentare, Nutrizione e Sanità Pubblica Veterinaria, Istituto Superiore di Sanità, Rome, Italy;; d Polo Scientifico, Laboratorio di Chimica Bioinorganica, Università degli Studi di Firenze, Florence, Italy;; e Istituto di Bioscienze e Biorisorse, CNR, Napoli, Italy

**Keywords:** Carbonic anhydrase, sulfonamide, inhibitor, *Vibrio cholerae*, antibacterials

## Abstract

We discovered novel and selective sulfonamides/amides acting as inhibitors of the α-carbonic anhydrase (CA, EC 4.2.1.1) from the pathogenic bacterium *Vibrio cholerae* (VchCA). This Gram-negative bacterium is the causative agent of cholera and colonises the upper small intestine where sodium bicarbonate is present at a high concentration. The secondary sulfonamides and amides investigated here were potent, low nanomolar VchCA inhibitors whereas their inhibition of the human cytosolic isoforms CA I and II was in the micromolar range or higher. The molecules represent an interesting lead for antibacterial agents with a possibly new mechanism of action, although their CA inhibition mechanism is unknown for the moment.

## Introduction

*Vibrio cholerae* is a Gram-negative bacterium responsible of cholera, a severe and watery form of diarrhoea. Cholera is contracted by ingestion of food or water contaminated with the pathogen, and particularly with the *V. cholerae* serogroups (O1 and O139) associated with the expression of the cholera toxin. Following a short incubation period, symptoms begin and severe fluid loss can lead to severe dehydration, electrolyte imbalance and, ultimately, death^1^. Precise estimates of the global burden of cholera remain a challenge because the majority of cases are not declared due to limitations, in some countries, in health surveillance management systems and fear of negative impact on trade and tourism. The number of reported cholera cases remains high over the last decade; WHO gave, for 2015, an account of 172,454 cases and 1304 deaths notified by 42 countries^2^, though the global estimates range between 1.3 and 4 million cases and between 21,000 and 143,000 deaths per year^3^. The treatment of cholera infections, is mainly focused on the re-hydration by using saline–glucose solutions that can be combined, in case of severe dehydration, with antibiotics in order to stabilise highly dehydrated patients and reduce the duration of illness^4^. Tetracycline and quinolones have been widely used, but numerous multidrug-resistant strains of *V. cholerae* have been isolated from both clinical and environmental settings, and as a consequence, the use of antibiotics had to be restricted^1^ and alternative target need to be identified in order to develop more effective and safe drugs for cholera treatment.

*V. cholerae* survives and multiplies in the upper small intestine where sodium bicarbonate, described as a potential inducer of virulence gene expression, is present at a high concentration^5^. Moreover, *V. cholerae* can increase cytosolic bicarbonate levels by means of the carbonic anhydrase (CA), a metalloenzyme that catalyzes the hydration of CO_2_ to produce HCO_3_^−^6. The first class of CA from the bacterial pathogen *V. cholerae* was described by our group; it is an α-CA, denominated VchCA that^7^, similar to the other α-CA, has three His ligands, which coordinate the Zn(II) ion crucial for catalysis. An active site residue transfers a proton from the water coordinated to the Zn(II) ion to the environment, forming zinc hydroxide that represents the nucleophilic species of the enzyme^7^. More in detail, the zinc hydroxide attacks the CO_2_, bound in a hydrophobic pocket near the metal ion, forming a labile intermediate where the bicarbonate is coordinated to the Zn(II) that readily reacts with an incoming water molecule, releasing the bicarbonate into solution^8^. On the basis of the role played by the bicarbonate ion as a virulence factor for *V. cholerae*, the VchCA has been suggested as a potential target for antibiotic development.

## Experimental

### Chemistry

#### Material and methods

All reagents, solvents, and deuterated were purchased from Sigma-Aldrich (Milan, Italy). Melting points were determined on Tottoli (Buchi) or Kofler apparatus and are uncorrected. Infrared spectra were recorded on a Spectrum One ATR Perkin Elmer FT-IR spectrometer, vibrational frequencies are given in ν, wave number (cm^−1^). Nuclear magnetic resonance (^1^H-NMR, ^13^C-NMR) spectra were acquired on AVANCE400 or AVANCE200 Bruker spectrometer, in DMSO, CD_3_OD or CDCl_3_ at 27 °C. Chemical shifts are reported in parts per million δ (ppm) relatively to TMS as internal reference, and the coupling constants (*J*) are expressed in Hertz (Hz). Splitting patterns are designated as follows: s, singlet; bs, broad singlet; d, doublet; t, triplet; q, quadruplet; and dd, double doublet. Mass spectra were recorded on a ThermoFinnigan LCQ Classic LC/MS/MS ion trap equipped with an ESI source and a syringe pump. Samples (10^−4^–10^−5 ^M in MeOH/H_2_O 90:10) were infused in the electrospray system at a flow rate of 5–10 μl min^−1^. Elemental analyses for C, H, and N were obtained by a PE 2400 (Perkin-Elmer) analyzer, and the analytical results were within ±0.4% of the theoretical values for all compounds.

Synthesis of sulfonamides **1–4:**
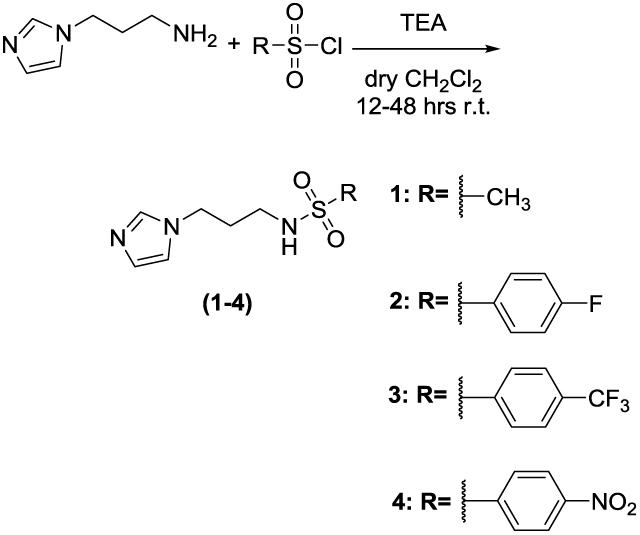


#### *N*-(3-(1*H*-imidazol-1-yl)propyl)methanesulfonamide 1

To a solution of 3-(1H-imidazol-1-yl)propan-1-amine (2.4 mmol) and TEA (3.6 mmol) in 3 ml of CH_2_Cl_2_, methanesulfonyl chloride (3.6 mmol) was added, and the mixture was kept overnight under stirring at room temperature. The solvent was evaporated under reduced pressure and the residue treated with a saturated solution of Na_2_CO_3_ (20 ml) and extracted in continuous for 24 h with CHCl_3_. After evaporation of the organic phase, **1** was purified by column chromatography on silica gel using CH_2_Cl_2_:MeOH (1:1) as mobile phase and obtained as a yellowish solid. M.p.: 89–91 °C (Tottoli-Buchi); 45% yield; ^1^H-NMR: δ_H_ (400 MHz, CDCl_3_) 7.52 (s, 1H), 7.09 (s, 1H), 6.97 (s,1H), 5.65 (bs, 1H), 4.12 (t, *J* = 6.5 Hz, 2H), 3.12–3.09 (m, 2H), 2.96 (s, 3H), 2.08 (m= 5, *J* = 6.5 Hz, 2H); ^13^C-NMR: δ_C_ (100 MHz, CDCl_3_): 137.4, 129.5, 118.8, 43.7, 39.97, 40.0, 31.5. MS-ESI^+^: *m*/*z* 203.93 [M + H]^+^.

#### *N*-(3-(1*H*-imidazol-1-yl)propyl)-4-fluorobenzenesulfonamide 2

To a solution of 3-(1H-imidazol-1-yl)propan-1-amine (0.8 mmol) and TEA (1.2 mmol) in 3 ml of CH_2_Cl_2_, 4-fluorobenzene-1-sulfonyl chloride (1.2 mmol) was added, and the mixture was kept overnight under stirring at room temperature. The reaction mixture was diluted with 5 ml of CH_2_Cl_2_ and washed with a saturated solution of NaHCO_3_ (5 ml). The aqueous phase was extracted with CH_2_Cl_2_ (2 × 5 ml), then the reunited organic layers were dried over Na_2_SO_4_, filtered and evaporated to give a residue that was purified by column chromatography on silica gel using as mobile phases firstly CH_3_CN and subsequently AcOEt:EtOH (1:1), **2** was obtained as a white solid. M.p.: 121–123 °C (Tottoli-Buchi); 40% yield; ^1^H-NMR: δ_H_ (400 MHz, DMSO-d_6_) 7.86–7.82 (m, 2H), 7.77 (bs, 1H), 7.55 (s, 1H), 7.47–7.43 (m, 2H), 7.09 (s, 1H), 6.86 (s, 1H), 3.96 (t, *J* = 7.0 Hz, 2H), 2.68 (t, *J* = 7.0 Hz, 2H), 2.51 (m = 5, *J*= 7.0 Hz, 2H); ^13^C-NMR: δ_C_ (100 MHz, DMSO-d_6_) 165.1 (*J* = 240.0 Hz), 137.2, 135.8 (*J* = 4.0 Hz), 129.8 (*J* = 9.0 Hz), 126.3, 119.5, 116.3 (22 Hz), 44.7, 39.5, 30.6; MS-ESI^+^: *m*/*z* 283.87 [M + H]^+^.

#### *N*-(3-(1*H*-imidazol-1-yl)propyl)-4-(trifluoromethyl)benzenesulfonamide 3

To a solution of 3-(1H-imidazol-1-yl)propan-1-amine (1.0 mmol) and TEA (1.5 mmol) in 5 ml of CH_2_Cl_2_, 4-(trifluoromethyl)benzene-1-sulfonyl chloride (1.5 mmol) was added, and the mixture was kept overnight under stirring at room temperature. The reaction mixture was washed with a saturated solution of Na_2_CO_3_ (3 × 5 ml). The organic layer, dried over Na_2_SO_4_, was evaporated and the residue was purified by column chromatography on silica gel using AcOEt:MeOH (8:2). The fractions with R_f_ = 0.21–0.40 were collected and purified using alumina and CH_2_Cl_2_:MeOH (9:1) to give a white residue that was left to solidify. M.p.: 127–128 °C (Kofler); 10% yield; ^1^H-NMR: δ_H_ (200 MHz, DMSO-d_6_) 8.06–7.98 (m, 5H), 7.67 (s, 1H), 7.14 (s, 1H), 6.92 (s, 1H), 3.96 (t, *J* = 6.4 Hz, 2H), 2.72 (t, *J* = 6.4 Hz, 2H), 2.08 (m = 5, *J* = 6.8 Hz, 2H); ^13^C-NMR: δ_C_ (100 MHz, DMSO-d_6_) 144.6, 137.6, 132.3 (*J* = 32.0 Hz), 128.3, 127.9, 127.0 (*J* = 3.7 Hz), 124.0 (*J* = 271.0 Hz), 119.9, 43.7, 40.07, 31.1; MS-ESI^+^: *m*/*z* 334.13 [M + H]^+^.

#### *N*-(3-(1*H*-imidazol-1-yl)propyl)-4-nitrobenzenesulfonamide 4

To a solution of 3-(1H-imidazol-1-yl)propan-1-amine (1.0 mmol) and TEA (1.5 mmol) in 5 ml of CH_2_Cl_2_, 4-nitrobenzene-1-sulfonyl chloride (1.5 mmol) was added, and the mixture was kept overnight under stirring at room temperature. The reaction mixture was washed with a saturated solution of Na_2_CO_3_ (3 × 5 ml). The organic layer, dried over Na_2_SO_4_, was evaporated and purified by column chromatography on silica gel using AcOEt:MeOH (8:2) as eluent, to give a white solid which was crystallised from AcOEt. M.p.: 140–141 °C (Kofler); 16% yield; ^1^H-NMR: δ_H_ (200 MHz, CD_3_OD) 8.41 (d, *J* = 9.1 Hz, 2H), 8.05 (d, *J* = 9.1 Hz, 2H), 7.62 (s, 1H), 7.10 (s, 1H), 6.95 (s, 1H), 4.10 (t, *J* = 6.8 Hz, 2H), 2.87 (t, *J* = 6.8 Hz, 2H),1.95 (m = 5, *J* = 6.8 Hz, 2H); ^13^C-NMR: δ_C_ (100 MHz, CD_3_OD) 150.1, 146.0, 137.1, 127.9, 127.8, 124.0, 119.2, 43.4, 39.3, 30.7; MS-ESI^+^: *m*/*z* 311.13 [M + H]^+^.

Synthesis of amides **5–9:**
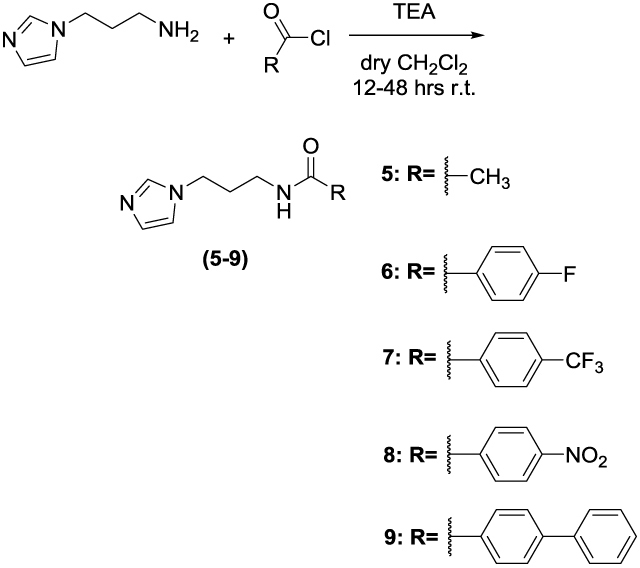


#### *N*-(3-(1*H*-imidazol-1-yl)propyl)acetamide 5

To a solution of 3-(1H-imidazol-1-yl)propan-1-amine (1.0 mmol) and TEA (1.5 mmol) in 5 ml of CH_2_Cl_2_, acetyl chloride (1.5 mmol) in 5 ml of CH_2_Cl_2_ was added dropwise. After 12 h at RT, the reaction mixture was evaporated under vacuum, the obtained residue was treated with a saturated solution of Na_2_CO_3_, and the aqueous solution was extracted in continuous with CHCl_3_ for 24 h. After solvent evaporation, the crude residue was purified by column chromatography on silica gel using CH_2_Cl_2_:MeOH (9:1) to give a colourless oil. IR ν: 1640 (C = O) cm^−1^; 65% yield; ^1^H-NMR: δ_H_ (400 MHz, CD_3_OD) 7.69 (s, 1H), 7.17 (s, 1H), 6.98 (s, 1H), 4.08 (t, *J* = 7.0 Hz, 2H), 3.18 (t, *J* = 7.0 Hz, 2H), 2.03–1.95 (m, 5H); ^13^C-NMR: δ_C_ (100 MHz, CDCl_3_) 170.8, 137.1, 128.9, 119.0, 44.8, 36.6, 31.0, 23.1; MS-ESI^+^: *m*/*z* 167.87 [M + H]^+^.

#### *N*-(3-(1*H*-imidazol-1-yl)propyl)-4-fluorobenzamide 6

To a solution of 3-(1H-imidazol-1-yl)propan-1-amine (1.0 mmol) and TEA (1.5 mmol) in 5 ml of CH_2_Cl_2_, 4-fluorobenzoyl chloride (1.5 mmol) was added. The reaction mixture was stirred for 12 h at RT and then washed with a saturated aqueous Na_2_CO_3_ (3 × 5 ml). The organic layer, dried over Na_2_SO_4_, was evaporated, and the obtained residue was purified by column chromatography on silica gel using CH_2_Cl_2_:MeOH (8:2) to give an oil which was solidified by treatment with petroleum ether M.p.: 70–72 °C (Tottoli-buchi); 60% yield. IR ν: 1650 (C = O) cm^−1^; ^1^H-NMR: δ_H_ (400 MHz, CD_3_OD) 7.90–7.86 (m, 2H), 7.72 (s, 1H), 7.23–7.18 (m, 3H), 6.99 (s, 1H), 4.14 (t, *J* = 7.0 Hz, 2H), 3.41 (t, *J* = 7.0 Hz, 2H), 2.12 (m = 5, *J* = 7.0 Hz, 2H); ^13^C-NMR: δ_C_ (100 MHz, CDCl_3_) 167.0, 164.7 (*J* = 250.0 Hz), 130.1, 130.4, 129.6 (*J* = 9.1 Hz), 128.6, 119.2, 115.4 (*J* = 21.0 Hz), 44.9, 37.1, 30.9; MS-ESI^+^: *m*/*z* 247.60 [M + H]^+^.

#### *N*-(3-(1*H*-imidazol-1-yl)propyl)-4-(trifluoromethyl)benzamide 7

To a solution of 3-(1H-imidazol-1-yl)propan-1-amine (0.8 mmol) and TEA (1.2 mmol) in 3 ml of CH_2_Cl_2_, 4-(trifluoromethyl)benzoyl chloride (1.2 mmol) was added, and the mixture was kept overnight under stirring at room temperature. The reaction mixture was diluted with 5 ml of CH_2_Cl_2_ and washed with a saturated aqueous NaHCO_3_ (5 ml). The aqueous phase was extracted with CH_2_Cl_2_ (2 × 5 ml), the combined organic layers were dried over Na_2_SO_4_, filtered and evaporated to give a residue that was purified by column chromatography on silica gel using CH_2_Cl_2_:MeOH (8:2). Compound **7** was obtained as a white solid. M.p.: 106–107 °C (Tottoli-Buchi); 80% yield; IR ν: 1657 (C = O) cm^−1^; ^1^H-NMR: δ_H_ (400 MHz, DMSO-d_6_) 8.75 (bs, 1H), 8.04 (d, *J* = 7.9 Hz, 2H), 7.86 (d, *J* = 7.9 Hz, 2H), 7.66 (s, 1H), 7.21 (s, 1H), 6.90 (s, 1H), 4.05–4.02 (m, 2H), 3.29–3.23 (m, 2H), 2.01–1.95 (m, 2H); ^13^C-NMR: δ_C_ (100 MHz, CD_3_OD) 167.6, 137.9, 137.1, 132.7 (q, *J* = 60.1 Hz), 127.8, 127.7, 125.1 (q, *J* = 10.2 Hz), 122.6, 119.2, 44.3, 36.9, 30.5; MS-ESI^+^: *m*/*z* 297.80 [M + H]^+^.

#### *N*-(3-(1*H*-imidazol-1-yl)propyl)-4-nitrobenzamide 8

To a solution of 3-(1H-imidazol-1-yl)propan-1-amine (0.8 mmol) and TEA (1.2 mmol) in 3 ml of dry CH_2_Cl_2_, 4-nitrobenzoyl chloride (1.2 mmol) was added, and the mixture was kept overnight under stirring at room temperature. The reaction mixture was then diluted with 5 ml of CH_2_Cl_2_ and washed with a saturated aqueous Na_2_CO_3_ (5 ml). The aqueous phase was extracted with CH_2_Cl_2_ (2 × 5 ml), and then the combined organic layers were dried over Na_2_SO_4_, filtered and evaporated under reduced pressure. The crude residue was purified by chromatography on silica gel using CH_2_Cl_2_:MeOH (9:1). The obtained yellowish solid was crystallised from CH_3_CN. M.p.: 152–154 °C (Tottoli-Buchi); 41% yield; IR ν: 1658 (C = O) cm^−1^; ^1^H-NMR: δ_H_ (400 MHz, DMSO-d_6_) 8.33 (d, *J* = 8.9 Hz, 2H), 8.02 (d, *J* = 8.9 Hz, 2H), 7.73 (s, 1H), 7.21 (s, 1H), 6.99 (s,1H), 4.16 (t, *J* = 6.9 Hz, 2H), 3.34 (t, *J* = 6.9 Hz, 2H), 2015 (m = 5, *J* = 6.9 Hz, 2H,); ^13^C-NMR: δ_C_ (100 MHz, CD_3_OD) 166.9, 149.6, 139.9, 137.2, 128.3, 127.8, 123.2, 119.2, 44.3, 37.0, 30.4; MS-ESI^+^: *m*/*z* 274.97 [M + H]^+^.

#### *N*-(3-(1*H*-imidazol-1-yl)propyl)-[1,1′-biphenyl]-4-carboxamide 9

To a solution of 3-(1H-imidazol-1-yl)propan-1-amine (1.0 mmol) and TEA (1.5 mmol) in 5 ml of dry CH_2_Cl_2_, biphenyl-4-carbonyl chloride (1.5 mmol) was added. The reaction mixture was kept under stirring at room temperature for 48 h, and then was washed with a saturated aqueous of Na_2_CO_3_ (3 × 5 ml). The organic layer, dried over Na_2_SO_4_, filtered and evaporated under reduced pressure. The crude residue was purified by column chromatography on silica gel using CH_2_Cl_2_:MeOH (9:1) to give a solid that was twice crystallised from AcOEt. M.p.: 135–136 °C; 26% yield. IR ν: 1639 (C = O) cm^−1^; ^1^H-NMR: δ_H_ (400 MHz, DMSO-d_6_) 8.57 (bs, 1H), 7.94 (d, *J* = 8.4 Hz, 2H), 7.78–7.72 (m, 4H), 7.67 (s, 1H), 7.51 (t, *J* = 7.2 Hz, 2H) 7.41 (t, *J* = 7.6 Hz, 1H), 7.22 (s, 1H), 6.90 (s, 1H), 4.03 (t, *J* = 6.8 Hz, 2H), 3.26 (q, *J* = 6.0 Hz, 2H), 1.98 (m =5, *J* = 6.8 Hz, 2H); ^13^C-NMR: δ_C_ (100 MHz, CD_3_OD) 168.7, 144.3, 139.8, 137.1, 132.8, 128.6, 127.8, 127.7, 127.5, 126.7, 126.6, 119.3, 44.4, 36.8, 30.7; MS-ESI^+^: *m*/*z* 306.07 [M + H]^+^.

Synthesis of 4-Nitro-*N*-(3-phenylpropyl)benzamide **10:**
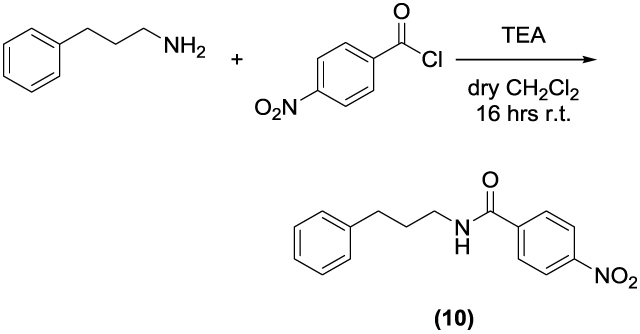


To a solution of 3-phenylpropan-1-amine (1.0 mmol) and TEA (1.5 mmol) in 5 ml of dry CH_2_Cl_2_, 4-nitrobenzoyl chloride (1.5 mmol) was added, and the mixture was kept overnight under stirring at room temperature. The reaction mixture was washed with a saturated aqueous of Na_2_CO_3_ (3 × 5 ml). The organic layer, was dried over Na_2_SO_4_, filtered and evaporated to give a white solid that was washed with petroleum ether and crystallised from toluene. M.p.: 95–97 °C (Tottoli-Buchi); 87% yield. IR ν: 1636 (C = O) cm^−1^; ^1^H-NMR δ_H_ (400 MHz, CD_3_OD) 8.33 (d, *J* = 7.9 Hz, 2H), 8.01 (d, *J* = 7.9 Hz, 2H), 7.30–7.15 (m, 5H), 3.45 (t, *J* = 6.9 Hz, 2H), 2.73 (t, *J* = 6.9 Hz, 2H), 1.98 (m = 5, *J* = 6.8 Hz, 2H); ^13^C-NMR: δ_C_ (100 MHz, CD_3_OD) 165.29, 149.51, 141.35, 140.16, 128.73, 128.41, 128.00, 126.26, 123.73, 40.31, 33.71, 30.80; MS-ESI^+^: *m*/*z* 283.07 [M + H]^+^.

### Carbonic anhydrase assay

A stopped-flow method^9^ was used for assaying the CA catalysed CO_2_ hydration activity with phenol red as indicator, working at the absorbance maximum of 557 nm, following the initial rates of the CA-catalyzed CO_2_ hydration reaction for 10–100 s. For each inhibitor, at least six traces of the initial 5–10% of the reaction have been used for determining the initial velocity. The uncatalyzed rates were determined in the same manner and subtracted from the total observed rates. Stock solutions of inhibitor (0.01 mM) were prepared in distilled–deionised water with 5% DMSO and dilutions up to 0.1 nM were done thereafter with the assay buffer. The Inhibition constant (*K*_I_) was obtained by considering the classical Michaelis–Menten equation, which has been fitted by non-linear least squares by using PRISM 3. All CA isozymes used in the experiments were purified recombinant proteins obtained as reported earlier by our group^10–18^.

### *In vitro* antibacterial assay

In order to evaluate the antibacterial activity of the synthesised compounds, an *in vitro* assay was performed using two *V. cholerae* O1 strains, the reference strain ATCC14103 (American Type Culture Collection; Manassas, VA) and ISS-Vc014, a clinical isolate from 1992 cholera outbreak in Luanda, Angola (courtesy of prof. Mauro Maria Colombo), previously characterised for a multiresistance profile (ampicillin, chloramphenicol, penicillin, streptomycin, spectinomycin, kanamycin, trimethoprim–sulfamethoxazole, tetracycline, and erythromycin)^19^,^20^. A procedure based on the method described in Andrews^21^ for broth dilution MICs was used for the tests: the studied compounds were dissolved in dimethyl sulfoxide (DMSO) and added to the liquid culture media (tryptic soy broth, Oxoid, Basingstoke, UK) in order to obtain final concentrations ranging from 0.1 μg ml^−1^ to 1 mg ml^−1^. The tests were performed in a microwell format (100 μl), and each strain was seeded at a concentration of 10^5^ c.f.u. ml^−1^; the inoculated plates were incubated without shaking at 37 °C for 18–20 h. Ampicillin (Sigma–Aldrich; Saint Louis, MO), tested in the same dilutions of the compounds, was used in the experiments as a reference drug, and a reference strain for MIC tests (*E. coli* ATCC 25922) was included to validate the results. A minimum of two wells containing uninoculated medium, medium inoculated with the dilutions of the compounds only, and medium inoculated with the bacterial strains and DMSO in amounts equivalent to those used to dissolve the compounds were also included in each test to act respectively as sterility and growth controls. Compounds showing at least partial inhibition of the bacterial growth at 1 mg ml^−1^ were selected to be tested at higher concentrations, and tests were performed with the above described procedure for concentration ranging from 1 mg ml^−1^ to 5 mg ml^−1^, with 0.5 mg ml^−1^ increments.

## Results and discussion

The rationale of this work was to design molecules, which may show enhanced affinity for CA from the bacterial pathogen *V. cholerae*, since in the last period many bacterial CA inhibitors were shown to possess interesting anti-infective action^22–26^. In addition, the imidazole ring is a group known for its ability to form coordination bonds, and there are many examples in the biological systems of complexes between the amino acid histidine and the zinc ion, although this type of binding has rarely been observed for CA inhibitors^27–30^. On these bases, we have assumed that the azole nitrogen could interact with the zinc ion or with the zinc ion coordinated water molecule^31–36^, blocking thus the enzyme activity, and therefore, a series of imidazole containing compounds was synthesised and evaluated as inhibitors of the bacterial enzyme VchCA; moreover, the ability to inhibit the human physiologically most important, CA I and II (hCA I and hCA II) was evaluated.

**Scheme 1.**
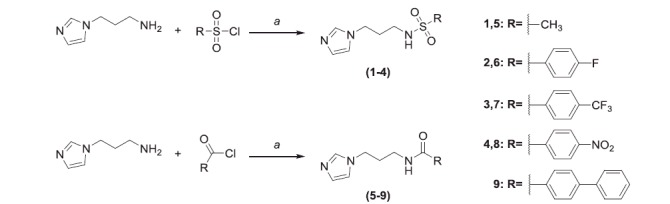
General synthesis of **1–9**. Reagents and conditions: (*a*): TEA, dry CH_2_Cl_2_, 12–48 h, RT.

**Scheme 2.**
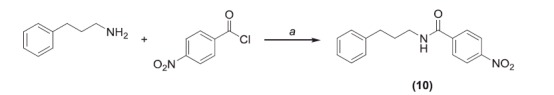
Synthesis of **10**. Reagents and conditions: (*a*): TEA, dry CH_2_Cl_2_, 16 h, RT.

Two set of amides and sulfonamides were synthesised (Scheme 1), including compound **10**, where the azole was replaced with a phenyl ring (Scheme 2). The latter compound was synthesised in order to demonstrate the possible involvement of the imidazole moiety in the inhibition of CA.

The synthesis of **1–9** was carried out by adding the appropriate sulfonylchloride or acyl chloride to a solution of 3-(1H-imidazol-1-yl)propan-1-amine and TEA in dry CH_2_Cl_2_ and stirring the reaction mixture at RT for 16–48 h. **10** was prepared from 3-phenylpropan-1-amine and 4-nitrobenzoyl chloride. All final compounds were purified by gravity column chromatography to afford products with high purity in yields between 10 and 87%; the analytical and spectroscopic data of the purified compounds are in agreement with the purposed structures. The detailed procedures for **1–10** are reported in the Supporting Information.

### Carbonic anhydrase inhibition

We assessed the CA inhibitory activity of compounds **1–10**, using the clinically acetazolamide (**AAZ**) as reference drug, for the inhibition of two human (h) isoforms, hCA I and II (cytosolic, widely distributed enzymes) as well as α-VchCA. The results are reported in Table 1.

**Table 1.** Inhibition activity of compounds **1–10** and **AAZ** against *Vibrio cholerae* α-carbonic anhydrase, obtained as described in the supplementary information.

		*K*_I_ (nM)^a^		
	Compound	hCA I	hCA II	VchCA
**1**		387.36	>10,000	10.9
**2**		1697.42	>10,000	8.5
**3**		>50,000	>50,000	6.4
**4**		>50,000	>50,000	6.2
**5**		>50,000	>50,000	39.2
**6**		>50,000	>50,000	9.1
**7**		2549.16	>10,000	10.4
**8**		1730.79	>10,000	11.0
**9**		>50,000	>50,000	5.4
**10**		>50,000	>50,000	7.8
**AAZ**		250	12.1	5.0

a Data represent the mean of 3 different assays; the mean errors are ±5–10% of the reported values.

Aromatic sulfonamide derivatives (**2–4**) exhibited inhibitory activity towards VchCA with inhibition constants ranging between 6.2 and 8.5 nM, comparable to that obtained by the simplest methyl derivative **1** and comparable or slightly higher than the **AAZ** (*K*_i_ = 5.0 nM). In the series of amides, the aromatic derivatives (**6–9**) generally showed a higher activity (*K*_i_ = 5.4–10.99 nM) than the acetoamide derivative **5** (*K*_i_ = 39.2 nM) with the most active compound being the biphenyl derivative **9**, that exhibited a *K*_i_ value of 5.4 nM, comparable to AAZ.

However, all synthesised compounds are selective inhibitor for the bacterial isoform over the human ones, hCA I and hCA II. Indeed, unlike the standard drug that is not selective, sulfonamides and amides reported here were generally ineffective inhibitors against hCA I, showing inhibition constants up to values higher 50,000 nM, and hCA II, on which no activity has been reported.

Surprisingly, the compound **10**, where a phenyl ring replaces the imidazole moiety of compound **8**, maintain the inhibitory activity towards VchCA, thus suggesting that the imidazole ring does not represents an essential requirement for the VchCA inhibition activity of these compounds. It is thus quite challenging to understand the CA inhibition mechanism with these compounds, which probably act by the so-called inhibition mechanism 5^37^, i.e. an unknown one. Several other classes of compounds, such as the secondary/tertiary sulfonamides and the protein tyrosine kinase inhibitors imatinib/nilotinib inhibit CAs by this mechanism of action which is not understood in details for the moment, since no X-ray crystal structures for adducts of the enzyme with such inhibitors could be obtained.

### The antibacterial activity

Based on the high inhibition activity observed in the enzymatic tests, we decided to evaluate *in vitro* the antibacterial activity of the synthesised compounds. Compounds were tested on two *V. cholerae* O1 strains of, the ATCC14103 and strain ISS-Vc014, a clinical isolate previously characterised for antibiotic resistance. Ampicillin, used as reference drug, showed inhibition activity at concentration higher than 0.01 mg ml^−1^ against the ATCC14103 strain and at 1.0 mg ml^−1^ against the ISS-Vc014 strain.

Among the ten compounds tested in concentrations ranging from 0.1 μg ml^−1^ to 1 mg ml^−1^, a partial inhibition of the bacterial growth was observed for compounds **2**, **7,** and **8** at the concentration of 1 mg ml^−1^, while no growth inhibition was observed for the other compounds. Compounds **2**, **7,** and **8** were then selected to be tested at higher concentrations (1 mg ml^−1^ to 5 mg ml^−1^). Compound **7** resulted not active until the concentration of 3 mg ml^−1^ (the limit of solubility), while compound 8 resulted partially active at the concentration of 3 mg ml^−1^ (the limit of solubility). Finally, compound **2** inhibited the bacterial growth of both *V. cholerae* ATCC14103 and ISS-Vc014 strain at a concentration of 2 mg ml^−1^. Compared to ampicillin, this compound resulted less active against the ATCC14103 strain, but possessed a comparable activity in the test on the strain ISS-Vc014.

Despite the compounds **2**, **7,** and **8** possess a high power on isolated VchCA, they resulted not particularly active in the cellular assay. This fact could be related to limited permeability of the cell wall to such compounds, leading to a reduced uptake into the cell, or to an efficient active efflux from the cell itself, a mechanism largely exploited by *V. cholerae* for antibiotic resistance^1^.

## Conclusions

In conclusion, on the basis of the role played by the bicarbonate ion as a virulence factor of *V. cholerae*, the VchCA could represent an interesting molecular target for the development of antibacterial drugs. For this purpose, we have synthesised a series of imidazole derivatives which were able to inhibit VchCA at nM concentrations; these compounds were also highly selective because they were inactive toward the human CA isoforms hCA I and II.

However, much work remains to be done, on one side to clarify the importance of the imidazole moiety in the enzyme inhibition processes, and on the other one to make these compounds more active both in the enzymatic and cellular assays, in order to identify novel antiinfective leads.
